# The impact of different 75 g oral glucose tolerance test target ranges within normal limits on neonatal outcomes: A validation study

**DOI:** 10.4274/tjod.40370

**Published:** 2018-03-29

**Authors:** Seda Subaş, Gökçe Anık İlhan, Zehra Meltem Pirimoğlu

**Affiliations:** 1 Dr. Lütfi Kırdar Kartal Training and Research Hospital, Clinic of Obstetrics and Gynecology, İstanbul, Turkey; 2 Marmara University Faculty of Medicine, Department of Obstetrics and Gynecology, İstanbul, Turkey

**Keywords:** Gestational diabetes mellitus, glucose tolerance test, neonatal outcome, screening, pregnancy

## Abstract

**Objective:**

To investigate the impact of different 75 g glucose tolerance test (OGTT) target ranges within normal limits on neonatal outcomes, thus to investigate the validity of 75 g OGTT thresholds.

**Materials and Methods:**

The normal 1-hour and 2-hour ranges of 75 g OGTT levels of 110 pregnant women with no gestational diabetes mellitus (GDM) were further divided into three different sub-groups; for the 1 hour as group 1 (<120 mg/dL), group 2 (120-140 mg/dL), group 3 (>140 mg/dL) and for the 2 hour as group 1 (<120 mg/dL), group 2 (120-135 mg/dL), and group 3 (>135 mg/dL).

**Results:**

For the 1-hour results, there was no statistically significant difference between groups in terms of age, body mass index, multiparity, neonatal hypoglycemia, hyperbilirubinemia, neonatal intensive care unit admission, birth weight, and LGT rates; however, the rate of small-for-gestational-age (SGA) infants was significantly higher in group 2 compared with those in group 3. For the 2-hour results, statistically similar results were found between the groups.

**Conclusion:**

A 2-hour 75 g OGTT has reliable threshold values for GDM screening. However, because there are still adverse neonatal outcomes in women with OGTT results below the current thresholds and the number of SGA fetuses is higher in the glucose range 120-140 mg/dL of the first hour, the validity of the 75 g OGTT thresholds still needs further investigation.

**PRECIS:** A 2-hour 75 g glucose tolerance test has reliable threshold values for gestational diabetes mellitus screening. However, the validity of the 75 g glucose tolerance test thresholds still needs further investigation.

## Introduction

Gestational diabetes, which affects 3% to 6% of all pregnancies, is an important issue that should be handled with specific treatment in addition to routine antenatal care to reduce the risks of maternal and perinatal morbidity^(1)^. It has been suggested that risks for adverse outcomes differ according to the single or combined thresholds selected^(2)^. The hyperglycemia and adverse pregnancy outcome (HAPO) study, pointed out the continuously increased risk between maternal glucose levels and adverse pregnancy outcomes even within ranges previously considered to be normal for pregnancy^(3)^. This study was the cornerstone for the diagnosis of gestational diabetes mellitus  (GDM), identified using the 75 g oral glucose tolerance test (OGTT) when any of the following plasma glucose values are exceeded: fasting, ≥5.1 mmol/L (92 mg/dL); 1 h, ≥10 mmol/L (180 mg/dL); and 2 h, ≥8.5 mmol/L (153 mg/dL)^(4)^. These cut-offs recommended by the International Association of the Diabetes and Pregnancy Study Groups (IADPSG)^(5)^, have been adopted by the World Health Organization (WHO)^(6)^ and the American Diabetes Association (ADA)^(4)^. As a result of new diagnostic criteria, the increase in the incidence of GDM and use of treatment modalities will be inevitable; however, considering the increasing rates of obesity and diabetes globally, the changes are updated, and recommended to reduce adverse outcomes^(4,5,7)^. On the contrary, in 2015, the National Institute for Health and Care Excellence (NICE) opted for a higher fasting glucose threshold [fasting ≥5.6 mmol/L (≥101 mg/dL), and/or 2 h ≥7.8 mmol/L (≥140 mg/dL)]^(8)^. 

The objective of this study was to investigate the validity of 75 g OGTT thresholds by evaluating the impact of different 75 g OGTT target ranges within normal limits on neonatal outcomes because the diagnostic dilemma on the most appropriate test for GDM and its thresholds is still ongoing.

## Materials and Methods

This is a cross-sectional study of 110 consecutive pregnant women who attended our outpatient antenatal clinic and were diagnosed as having no GDM using the 75 g OGTT at 24-28 weeks of gestation. A 2 hour 75 g OGTT is performed for screening GDM at 24-28 weeks of gestation as a standard obstetric practice at our institution. The study protocol was approved by the Ethics Committee of Lütfi Kırdar Kartal Training and Research Hospital (89513307/1009/372). Written informed consent was obtained from all subjects before the study. The diagnosis of the GDM was made according to the ADA/IADPSG criteria, when any of the following plasma glucose values were exceeded: fasting, ≥92 mg/dL; 1 h, ≥180 mg/dL; 2 h, ≥153 mg/dL^(4,5)^. The exclusion criteria included women with GDM, pre-gestational diabetes mellitus (GDM), hypertension, multiple pregnancies, and fetal anomalies. The normal 1 h and 2 h ranges of 75 g OGTT levels of 110 pregnant women were further divided into three different sub-groups; for the 1 h as group 1 (<120 mg/dL), group 2 (120-140 mg/dL), group 3 (>140 mg/dL), and for the 2 h as group 1 (<120 mg/dL), group 2 (120-135 mg/dL), and group 3 (>135 mg/dL). Neonatal outcomes were compared between these new range groups. Neonatal hypoglycemia, hyperbilirubinemia, intensive care unit admission, large-for-gestational-age (LGA) and small-for-gestational-age (SGA) newborns were considered as adverse outcomes. The presence of one or more adverse outcome was determined as an abnormal result. 

### Statistical Analysis

All data were analyzed using SPSS Statistics for Windows, Version 22 (IBM Corp, Armonk, NY) and p values <0.05 were considered to be statistically significant. Continuous variables are presented as mean ± standard deviation and categorical variables as numbers and percentages. For the analysis of qualitative data, the chi-square test was used. For the analysis of quantitative data, One-Way ANOVA and Kruskal-Wallis tests were used.

## Results

One hundred ten pregnant women without GDM were enrolled in the study. The women were further divided into subgroups according to different ranges of normal 75 g OGTT results to compare neonatal outcomes.

The number and percentage of the subjects were 50 (45.5%), 32 (29%) and 28 (25.5%) for the first hour (Table 1), and 82 (74.5%), 14 (12.7%), and 14 (12.7%) (Table 2) for the second hour, for groups 1-3, respectively. For the 1 h results, there was no statistically significant difference between the groups in terms of age, body mass index (BMI), multiparity, neonatal hypoglycemia, hyperbilirubinemia, intensive care unit admission, birth weight, abnormal results, and LGA rates; however, the rate of SGA infants was statistically significantly higher in group 2 compared with group 3 (Table 1). For the 2 h results, statistically similar results were found between the groups (p>0.05) (Table 2). 

## Discussion

The accurate diagnosis of GDM and prompt and proper precautions to prevent adverse outcomes are crucial for both the mother and the fetus. There are many studies in the literature about the adverse effects of gestational diabetes on pregnancy outcomes^(3,9,10,11,12,13)^. The initial criteria for the diagnosis was determined more than 40 years ago^(14)^; however, the ongoing debate about the thresholds of the OGTT is yet to be concluded. The HAPO study, with a large, multinational cohort of 25505 pregnant women, showed a continuous relationship between maternal glycemia and adverse outcomes, with no obvious thresholds at which risks increased^(3)^. With the results showing a strong and continuous association between adverse outcomes and higher levels of maternal glucose, which are lower than those diagnostic of diabetes, and with the inclusion of a large number of subjects from a broad geographic area of the participating centers; this study changed the concept, and was the basis for the IADPSG new criteria, which was also adopted by WHO and ADA^(4,7)^. Considering the continuous relationship between glycemia and adverse outcomes, in our study, we investigated different 75 g OGTT target ranges within normal limits on neonatal outcomes and found adverse outcomes even in pregnant women with no GDA. The 2 h results were similar among groups in terms of age, BMI, multiparity, neonatal hypoglycemia, hyperbilirubinemia, intensive care unit admission, birth weight, abnormal result, SGA and LGA rates; however, for the 1 h results, the rate of SGA infants was statistically significantly higher in group 2 (120-140 mg/dL), compared with group 3 (>140 mg/dL). The American College of Obstetricians and Gynecologists reported that the one-step approach would increase the prevalence of GDM and health care costs without evidence for clinical improvements in maternal and neonatal outcomes, and favored the two-step approach^(15)^. In a recent study, it has been suggested that the one-step method identifies high-risk women at least as well as the two-step method^(16)^. Identifying subjects at risk and prompt, specific interventions to reduce maternal hyperglycemia can reduce maternal and perinatal morbidity^(1,17,18)^. In this present study, the 75 g OGTT one-step approach was used, and to minimize the risk the neonatal outcomes, the results were compared between different ranges, within the normal limits of the IADPSG/ADA criteria. In 2015, NICE recommended new diagnostic thresholds for the diagnosis of GDM, with a higher fasting but lower 2 h post-load glucose thresholds of those proposed by the IADPSG^(8)^. In a study to identify ethnic-specific criteria for the diagnosis of GDM, it was suggested that the United Kingdom NICE might have underestimated the prevalence of gestational diabetes, especially in south Asian women^(19)^. In another recent study that evaluated neonatal and obstetric outcomes among women who were test positive for the IADPSG criteria but negative for the NICE 2015 criteria, a higher risk for LGA, cesarean delivery, and polyhydramnios was suggested compared with women with negative screening results and no OGTT. The IADPSG criteria was determined to identify women at substantial risk of complications who would not be identified by the NICE 2015 criteria. As a result, it was reported that according to the NICE criteria, a high-risk group could be unidentified and left untreated depending on the higher fasting threshold, and a low-risk group could be treated instead depending on the lower 2 h threshold.^(20)^

### Study Limitations

The limitation of the study is its small sample size. The validity of the 75 g OGTT thresholds still needs to be investigated and verified by large studies.

## Conclusion

This study demonstrates that the 75 g OGTT (IADPSG/ADA) has reliable threshold values for GDM screening as the neonatal outcomes do not differ between the low normal and high normal levels of the first and second-hour test results, and provides evidence that there are still adverse neonatal outcomes in women with OGTT results below the current thresholds. The study also reports a higher number of SGA in the glucose range 120-140 mg/dL of the first hour, which needs further evaluation. As a result, the validity of the 75 g OGTT thresholds still needs to be investigated and verified by large studies.

## Figures and Tables

**Table 1:**
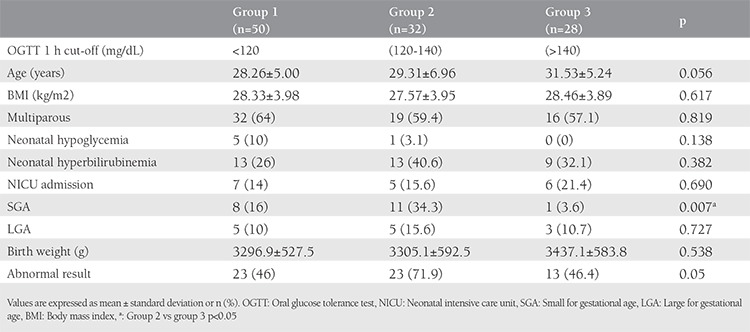
Maternal characteristics and neonatal outcomes of groups according to different 75 g oral glucose tolerance test target 1 h ranges within normal limits

**Table 2:**
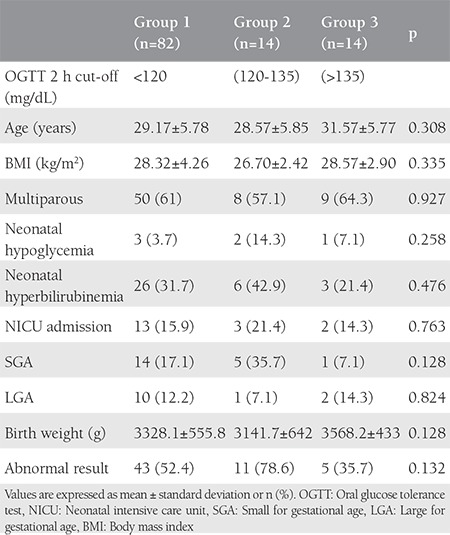
Maternal characteristics and neonatal outcomes of groups according to different 75 g oral glucose tolerance test target 2 h ranges within normal limits

## References

[1] Alwan N, Tuffnell DJ, West J (2009). Treatments for gestational diabetes. The Cochrane Database of Systematic Reviews.

[2] Black MH, Sacks DA, Xiang AH, Lawrence JM (2010). Clinical outcomes of pregnancies complicated by mild gestational diabetes mellitus differ by combinations of abnormal oral glucose tolerance test values. Diabetes Care.

[3] Metzger BE, Lowe LP, Dyer AR, Trimble ER, Chaovarindr U, Coustan DR, et al, HAPO Study Cooperative Research Group (2008). Hyperglycemia and adverse pregnancy outcomes. N Engl J Med.

[4] American Diabetes Association (ADA) (2011). Diagnosis and classification of diabetes mellitus. Diabetes Care.

[5] Metzger BE, Gabbe SG, Persson B, Buchanan TA, Catalano PA, Damm P, et al (2010). International association of diabetes and pregnancy study groups recommendations on the diagnosis and classification of hyperglycemia in pregnancy.International Association of Diabetes and Pregnancy Study Groups Consensus Panel. Diabetes Care.

[6] (2014). Diagnostic criteria and classification of hyperglycaemia first detected in pregnancy: a World Health Organization Guideline. World Health Organization (WHO). Diabetes Res Clin Pract.

[7] American Diabetes Association (ADA) (2016). Classification and Diagnosis of Diabetes. Diabetes Care.

[8] National Institute for Health and Care Excellence (NICE). Diabetes in pregnancy: management of diabetes and its complications from preconception to the postnatal period. Clinical guideline NG3.2015..

[9] Ovesen PG, Jensen DM, Damm P, Rasmussen S, Kesmodel US (2015). Maternal and neonatal outcomes in pregnancies complicated by gestational diabetes a nation-wide study. J Matern Fetal Neonatal Med.

[10] Laafira A, White SW, Griffin CJ, Graham D (2016). Impact of the new IADPSG gestational diabetes diagnostic criteria on pregnancy outcomes in Western Australia. Aust N Z J Obstet Gynaecol.

[11] Martino J, Sebert S, Segura MT, García-Valdés L, Florido J, Padilla MC, et al (2016). Maternal Body Weight and Gestational Diabetes Differentially Influence Placental and Pregnancy Outcomes. J Clin Endocrinol Metab.

[12] Peixoto AB, Caldas TM, Santos RO, Lopes KS, Martins WP, Araujo Júnior E (2016). The impact of gestational diabetes and hypothyroidism on the third-trimester ultrasound parameters and in adverse perinatal outcomes: a retrospective cohort study. J Matern Fetal Neonatal Med.

[13] Wu ET, Nien FJ, Kuo CH, Chen SC, Chen KY, Chuang LM, et al (2016). Diagnosis of more gestational diabetes lead to better pregnancy outcomes: Comparing the International Association of the Diabetes and Pregnancy Study Group criteria, and the Carpenter and Coustan criteria. J Diabetes Investig.

[14] O’sullıvan JB, Mahan CM (1964). Criteria for the oral glucose tolerance test in pregnancy. Diabetes.

[15] American College of Obstetricians and Gynecologists (ACOG) (2013). Practice Bulletin No. 137: Gestational diabetes mellitus. Obstetrics and Gynecology.

[16] March MI, Modest AM, Ralston SJ, Hacker MR, Gupta M, Brown FM (2016). The effect of adopting the IADPSG screening guidelines on the risk profile and outcomes of the gestational diabetes population. J Matern Fetal Neonatal Med.

[17] Crowther CA, Hiller JE, Moss JR, McPhee AJ, Jeffries WS, Robinson JS, Australian Carbohydrate Intolerance Study in Pregnant Women (ACHOIS) Trial Group (2005). Effect of treatment of gestational diabetes mellitus on pregnancy outcomes. N Engl J Med.

[18] Moss JR, Crowther CA, Hiller JE, Willson KJ, Robinson JS, Australian Carbohydrate Intolerance Study in Pregnant Women Group (2007). Costs and consequences of treatment for mild gestational diabetes mellitus - evaluation from the ACHOIS randomised trial. BMC Pregnancy Childbirth.

[19] Farrar D, Fairley L, Santorelli G, Tuffnell D, Sheldon TA, Wright J, et al (2015). Association between hyperglycaemia and adverse perinatal outcomes in south Asian and white British women: analysis of data from the Born in Bradford cohort. Lancet Diabetes Endocrinol.

[20] Meek CL, Lewis HB, Patient C, Murphy HR, Simmons D (2015). Diagnosis of gestational diabetes mellitus: falling through the net. Diabetologia.

